# Classification of fungal genera from microscopic images using artificial intelligence

**DOI:** 10.1016/j.jpi.2023.100314

**Published:** 2023-04-23

**Authors:** Md Arafatur Rahman, Madelyn Clinch, Jordan Reynolds, Bryan Dangott, Diana M. Meza Villegas, Aziza Nassar, D. Jane Hata, Zeynettin Akkus

**Affiliations:** a Department of Laboratory Medicine and Pathology (DLMP), Mayo Clinic, Jacksonville, Florida, USA; b Division of Clinical Microbiology, Mayo Clinic, DLMP, Jacksonville, Florida, USA; c Computational Pathology and Artificial Intelligence, DLMP, Mayo Clinic, Jacksonville, Florida, USA; d Department of Mathematics, Florida State University, Tallahassee, Florida, USA; e Department of Statistics, Florida State University, Tallahassee, Florida, USA

**Keywords:** Fungal genera classification, Artificial intelligence, Mycology, Convolutional neural network

## Abstract

Microscopic image examination is fundamental to clinical microbiology and often used as the first step to diagnose fungal infections. In this study, we present classification of pathogenic fungi from microscopic images using deep convolutional neural networks (CNN). We trained well-known CNN architectures such as DenseNet, Inception ResNet, InceptionV3, Xception, ResNet50, VGG16, and VGG19 to identify fungal species, and compared their performances. We collected 1079 images of 89 fungi genera and split our data into training, validation, and test datasets by 7:1:2 ratio. The DenseNet CNN model provided the best performance among other CNN architectures with overall accuracy of 65.35% for top 1 prediction and 75.19% accuracy for top 3 predictions for classification of 89 genera. The performance is further improved (>80%) after excluding rare genera with low sample occurrence and applying data augmentation techniques. For some particular fungal genera, we obtained 100% prediction accuracy. In summary, we present a deep learning approach that shows promising results in prediction of filamentous fungi identification from culture, which could be used to enhance diagnostic accuracy and decrease turnaround time to identification.

## Introduction

In the diagnostic mycology laboratory, microscopic examination and interpretation of fungal cultures continues to be the gold-standard for identification. Final identification can be highly subjective, dependent upon the skill of the laboratory technologist. Unsporulated hyphae and similar morphologies may contribute to misidentifications. Application of a deep learning approach may have value in the initial identification and classification of filamentous fungi from culture, with potential to increase diagnostic accuracy and decrease time to result.

Diseases caused by fungi occur on different parts of the human body such as skin, nails, mouth, and hair. According to the CDC, 75 000 hospitalizations and 9 million outpatient visits per year are due to mycoses.^1^, ^2^, ^3^, ^4^, ^5^ The current method for diagnosis of these fungal infections used by many laboratories is microscopic examination.^6^, ^7^, ^8^, ^9^ However, final diagnosis may be inconclusive due to the similar morphologies of different genera and among individual species within a genus. Biochemical tests, selective fungal culture media, and extended incubation may be required to obtain the correct identification. Fungal proteomic analysis and genetic sequencing are accurate but not available in many diagnostic laboratories. Laboratory evaluation can be costly and time consuming causing a delay in necessary treatment of the infection. Application of a deep learning algorithm used to classify mycologic images has the potential to increase accuracy and decrease both turnaround time and cost of diagnosis.

In the literature, there are currently few deep learning studies related to the diagnosis of fungal infections using microscopic images.^10^, ^11^ There are 2 different approaches we have seen so far. One study focuses on detecting hyphae, which is defining a structure of fungal infections.^7^ Another study focuses on classifying the infections based on microscopic morphology.^11^ Koo et al.^10^ presented automated detection of superficial fungal infections (i.e., hyphae) from microscopic images with 40x and 100x magnifications using an object detection convolutional neural network (CNN). They used a total of 38 samples with 18 positive cases and 20 negative cases to train and test the model. A YOLO v4 object detection CNN^12^ is trained to perform object detection of hyphae. Using probabilities and a threshold an automated decision is made to classify the image as positive (containing hyphae) or negative (not containing hyphae). This study was able to detect hyphae in microscopic images with high accuracy.

Zielinski et al.^11^ presented a deep learning approach to describe and classify fungi microscopic images. They used a machine learning method that is based on CNN and bag-of-words approach for classifying microscopic fungal images. The dataset used in this paper consists of 9 different fungal species with 180 images in total. With a limited amount of data, they found that fine tuning well-known architectures did not produce optimal results. The method that they present uses well-known architectures pretrained on ImageNet^13^ data to obtain the features’ block. Then points from the features’ block are pooled using Bag of Words or Fisher Vector. Finally, classification is done with either Support Vector Machine or Random Forest.^14^ Due to the small size of the dataset, they performed patch-based classification of size 500x500 pixels and then determined the most frequent prediction to obtain classification of the entire image scan. Based on their results, the AlexNet model, Fisher Vector pooling, and Support Vector Machine classification produced the most accurate results with a patch-based classification accuracy of 82.4% and a scan-based classification accuracy of 93.9%. In addition to these 2 researches, there is a review study done by Smith and Kirby^15^ where they discussed different approaches to classify microbial images using AI. They have shown the pros and cons of different AI-based methods in clinical microbiology.

**Fig. 1:**
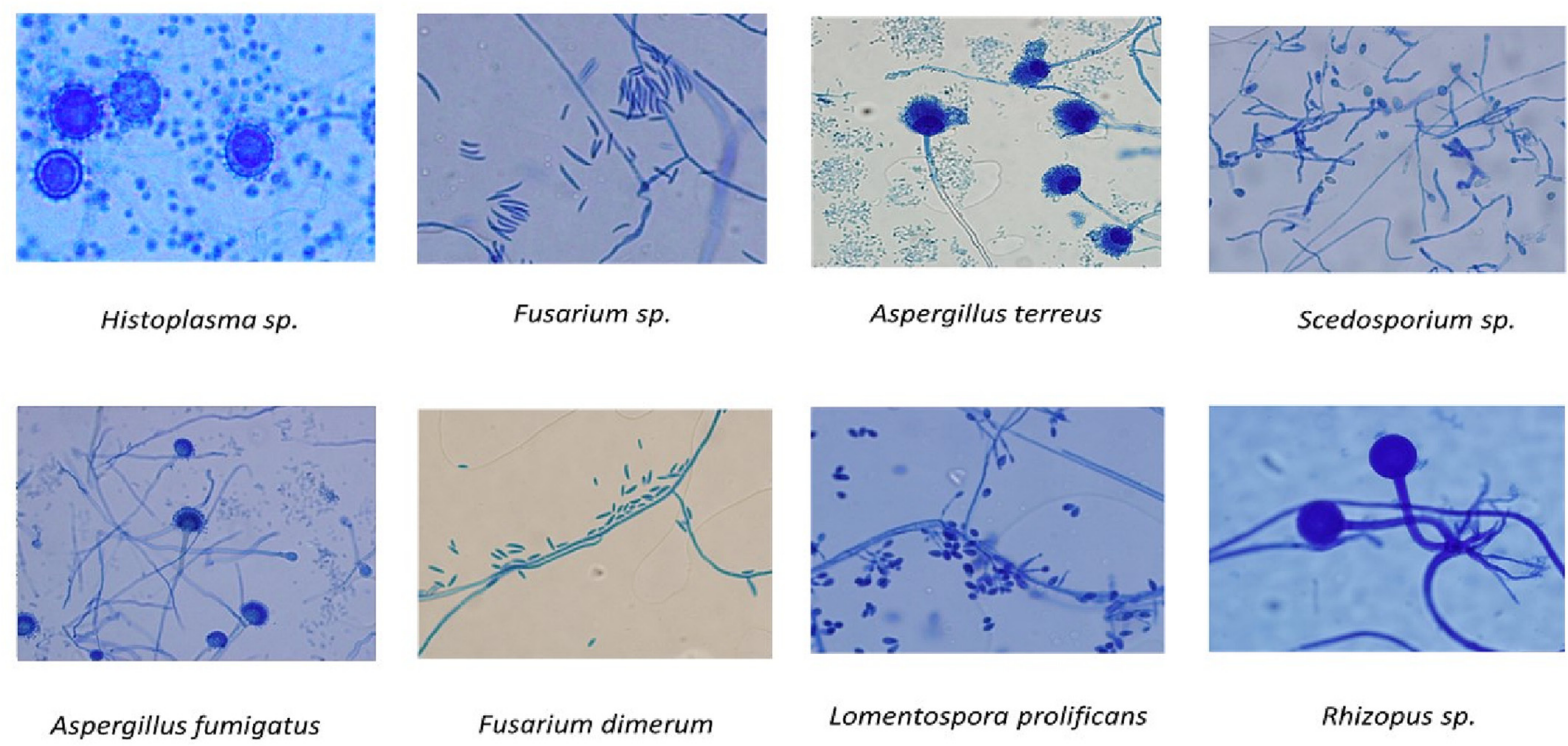
Microscopic images of some prevalent genera.

A limitation of current studies is that the models have only been trained and tested using a small amount of data for a limited number of species. There are over 600 fungal genera associated with humans.^9^, ^16^, ^17^, ^18^, ^19^, ^20^, ^21^, ^22^, ^23^, ^24^, ^25^, ^26^, ^27^, ^28^, ^29^, ^30^, ^31^, ^32^, ^33^ To be effective in classifying a larger variety of fungal genera, the dataset for training needs to contain a larger variety of images. Our study contributes to the current state of this field of study as we have trained a model with a more diverse dataset consisting of 89 clinically relevant fungal genera, focusing on human pathogens and examples of the most prevalent genera as shown in Fig. 1. Our study is designed as follows: Construction of our research model with a brief description of our dataset preparation. We then described our AI model and loss function followed by an analysis of results.

## Model description

**Fig. 2:**
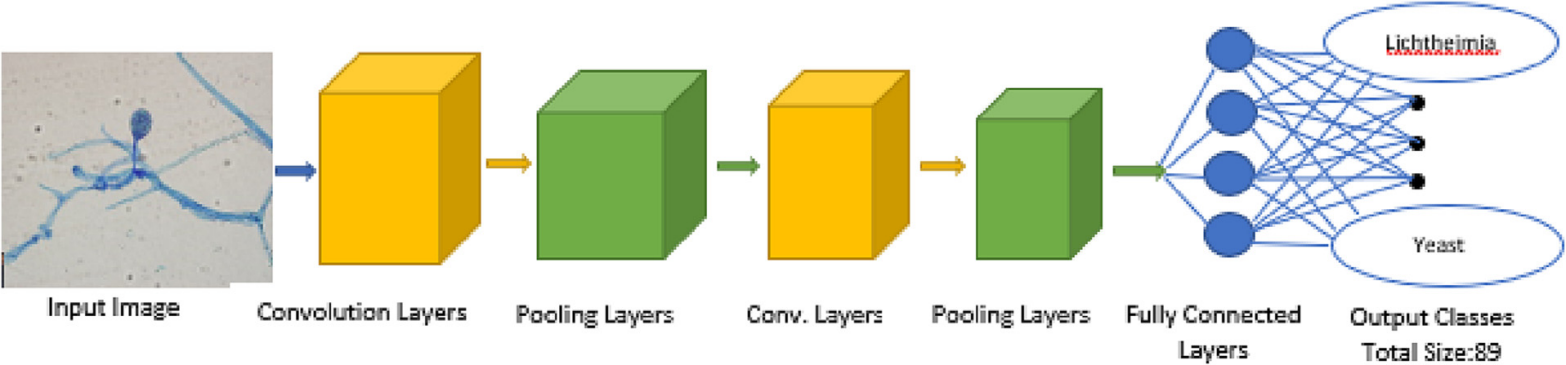
A general CNN architecture for fungal image classification.

Convolutional neural networks (CNNs) are very successful at identifying images.^17^, ^18^, ^19^, ^20^ We have used different versions of CNNs as our AI model. In a standard CNN, we send an input image to the model which passes through several convolution and pooling layers. Each layer takes the output of the previous layer as an input. These layers are used to identify different distinguishing features from the images. There are some activation functions inside those layers which bring nonlinearity in our model. At the end, there is a fully connected ‘Softmax’ layer which is used for multi-class image classification. A standard CNN architecture is given in Fig. 2.

The prediction in the output layers is a probability vector over 89 classes. For top 1 accuracy, our predicted class is the class with the highest probability. For top 3 accuracy, our predicted class is in any of the classes with the highest 3 probability.

Besides the architecture, our models have several other components. One important part is the loss function. When we train an AI model, we need to set a penalty for imbalanced classes. We minimize that loss function to update our model parameter at every step in our training and with sufficient amount of training we can get the optimal set of parameters. Those parameters are then used to make predictions on testing and validation data. Since we are solving a multi-class classification problem, we have used weighted categorical cross entropy as our loss function.^21^, ^22^ The mathematical formula for the categorical cross-entropy is given by

$$\mathrm{Loss}=-\sum_{i=1}^{N}y_{i}*\log\left({\hat{y}}_{i}\right)$$

Where *N* is the output size which is the number of scalar values in the model output, ${\hat{y}}_{i}$ is the i-th scalar value in the model output and *y*_*i*_ is the corresponding target value. The minus sign indicates that the loss gets smaller when the predicted and target values get closer to each other.

In addition, we also have other hyperparameters such as learning rate, early stopping criteria, optimizers, number of epochs, batch size etc. We optimized those parameters to improve model performance.

## Methods

**Fig. 3:**
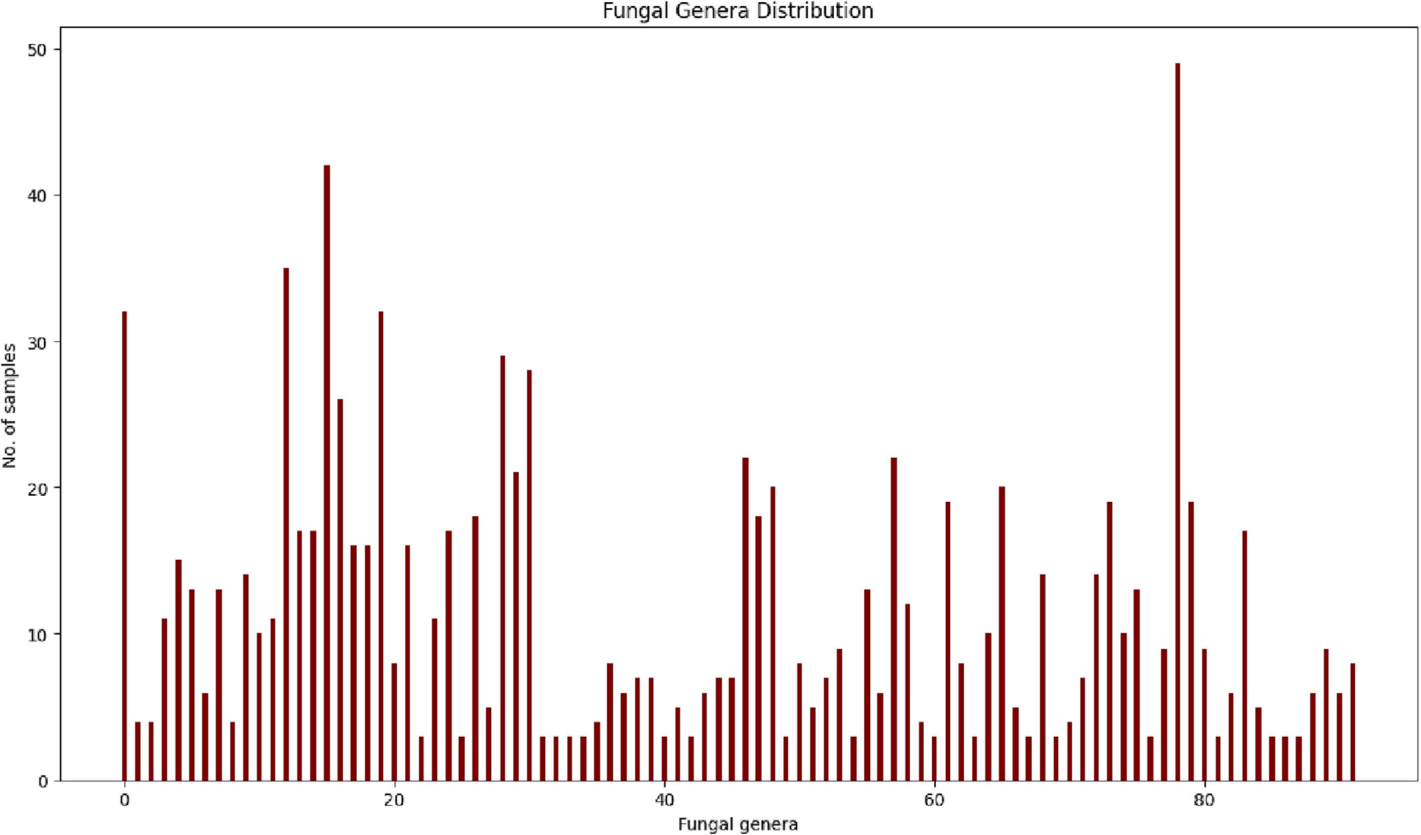
Distribution of 89 classes of fungal genera.

In this study, the RGB images of 89 fungal genera were collected as shown in Table 1. The distribution of samples for 89 classes is shown in Fig. 3. The total magnification of the images is 400X. That is the standard for the microscopic identification of fungi. The dimensions of the images are a function of the camera software we use—the images are uniform. On the original images, the software places a ruler so that we can estimate size in microns.The total number of sample images was 1079. We trained well-known convolutional neural networks (CNN) such as DenseNet^23^ Inception ResNet,^24^ InceptionV3,^25^, ^26^, ^34^ Xception,^28^, ^35^ ResNet50,^27^ VGG16, and VGG19^13^ to identify fungal genera and assess their performance. Genus specific data was split into train, test and validation sets by maintaining the ratio 7:2:1. The number of sample images in train, test, and validation sets were 668, 247, and 164 images, respectively.To increase sample number, we augmented our training data by processing our original images with 10 different random transformations which includes random rotations, translations, shearing in horizontal and vertical directions, flipping left-right, flipping up-down, resizing with pad or crop, adjusting brightness and contrast etc.

**Table 1:** The list of fungal genera used in this study.

*Candida auris*	*Cladophialophora* sp.	*Microsporum* sp*.*	*Scedosporium* sp.
*Candida parapsilosis*	*Cladosporium* sp.	*Microsporum canis*	*Scopulariopsis brevicaulis*
*Lomentospora* sp*. (Absidia)*	*Coccidioides* sp.	*Mucor* sp.	*Scopulariopsis brumptii*
*Acremonium* sp.	*Cryptococcus* sp.	*Biatriospora (Nigrograna) mackinnonii*	*Scytalidium* sp.
*Alternaria* sp.	*Cunninghamella* sp.	*Ochroconis* sp.	*Sporothrix* sp.
*Arthrographis* sp*.*	*Curvularia* sp.	*Phialophora verrucosa*	*Sporotrichum* sp.
*Aspergillus calidoustus*	*Drechslera* sp.	*Paecilomyces* sp.	*Stachybotrys* sp.
*Aspergillus clavatus*	*Emmonsia* sp.	*Paecilomyces variotii*	*Syncephalastrum* sp.
*Aspergillus flavus*	*Epidermophyton* sp.	*Penicillium* sp.	*Trichophyton* sp.
*Aspergillus fumigatus*	*Exophiala* sp.	*Pestalotia* sp.	*Trichophyton mentagrophytes*
*Aspergillus glaucus*	*Exophiala (Wangiella) dermatatiditis*	*Phialemoniopsis* sp.	*Trichophyton tonsurans*
*Aspergillus nidulans*	*Exserohilum* sp.	*Phialemonium* sp.	*Trichosporon* sp.
*Aspergillus niger*	*Fonsecaea* sp.	*Phialophora* sp.	*Trichosporon mucoides*
*Aspergillus* sp.	*Fonsecaea pedrosi*	*Phoma* sp.	*Ulocladium* sp.
*Aspergillus terreus*	*Fusarium* sp.	*Pithomyces* sp.	*Verticillium* sp.
*Aspergillus ustus*	*Fusarium dimerum*	*Pleurostomophora richardsiae*	Yeast not specified
*Aspergillus versicolor*	*Geotrichum* sp.	*Prototheca* sp.	
*Aureobasidium* sp.	*Graphium (P. boydii)*	*Pseudallescheria boydii (*formerly *S. apiospermum)*	
*Beauveria* sp.	*Histoplasma capsulatum*	*Rhinocladiella* sp.	
*Bipolaris* sp.	*Hormographiella* sp.	*Rhizopus* sp.	
*Chaetomium* sp.	*Hortaea werneckii*	*Sporothrix schenckii*	
*Chrysosporium* sp.	*Malassezia* sp.	*Scedosporium apiospermum*	
*Cladophialophora bantiana*	*Malbranchea* sp.	*Scedosporium prolificans*

The reference gold-standard for fungal identification in this study is fungal culture, followed by macroscopic and microscopic morphology performed by an experienced medical mycologist, with a review by a second mycologist. This is the standard for most clinical microbiology laboratories and is cited by regulatory bodies overseeing the lab. To evaluate our AI model performance, we considered the accuracy metric which was calculated by dividing the number of correctly predicted samples by the total number of samples. Then, we multiplied this ratio by 100 to obtain a percentage. We have also calculated precision, recall, and F1-score metrics to evaluate our results.

## Experiments

The success of an AI model depends on the nature of the training environment. To make our model more robust, we performed multiple training experiments by considering different criteria. The training portion is categorized into 2 types. Training with the original data and training with the augmented data. While training with the augmented data, we used original data plus 2 augmented datasets obtained from those random transformations mentioned in the previous section. Each augmented dataset had the same number of samples as the original dataset. For both types of training (original and augmented), we performed experiments by changing different parameters. We used both types of poolings Max and Average and different weight initializations such as ImageNet pretrained weight and Random weights.

In addition, we also performed experiments with transfer learning and fine tuning to check whether model performance was improved. For fine tuning, we made all layers non-trainable except the last 2 classifications layers and trained our models with ImageNet pretrained weights.

To assess the generalization ability of our best model, we applied 3-fold cross validation. We split our original samples into 3 folds. During the split, each fold contained at least 1 sample from each fungal genus. At that point, the DenseNet model was trained with the best parameters on any 2 folds, with validation of the prediction accuracy on the remaining fold. In this 3-fold cross validation, 2 different combinations of results were noted. We reported test accuracy in the experiments and used the paired samples t-test method in R for statistical comparisons of our results.

## Results

The ‘DenseNet’ CNN model gave the best performance among other CNN models with overall accuracy of 65.35% top 1 prediction and 75.19% accuracy for top 3 predictions. The precision, recall, and F1 score for top 1 predictions from the model DenseNet that were 65.35%, 75.11%, and 69.98% respectively. Those metrics were calculated by using “sci-kit-learn” precision, recall, and F1 score packages. Accuracy scores were increased by 10% when we added augmented data during the training. We also observed that if we trained our model with ‘ImageNet’ pretrained initial weights then accuracy scores improved roughly by 15%. There was not any significant improvement in prediction accuracy if Average pooling rather than Max pooling was used. The results from transfer learning and fine tuning were suboptimal. The test accuracy dropped below 50% in those cases. In cross validation, the test accuracy score for Fold1, Fold2, and Fold3 were 69.08%, 70.57%, and 69.32% respectively. Fold validation results were obtained from the model DensenNet by using the augmented data and ImageNet pretrained initialization for training.

**Fig. 4:**
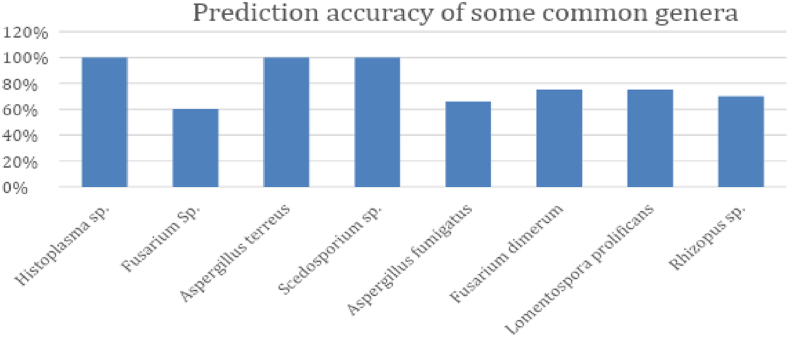
Prediction accuracy of some prevalent genera.

**Fig. 5:**
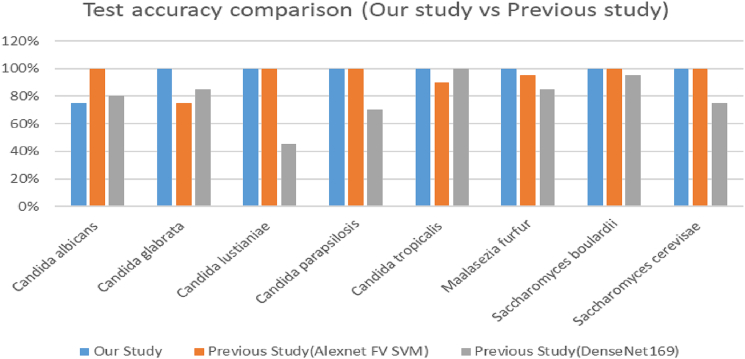
Test accuracy comparison between our study and previous study for some genera used in the previous study.^8^ Previous study results are for the model “AlexNet FV SVM” and “Densenet 169” in Zielinski et al.^11^

The results from the experiments are shown in the figures and table below. In Fig. 4, we have shown the prediction accuracy for prevalent genera was higher than the overall test prediction accuracy. For the genera *Histoplasma, Aspergillus, Scedosporium* nearly 100% test prediction was noted. Prediction results comparison between our study and the study in Zielinski et al^8^ for some yeast genera were shown in Fig. 5. Nearly 100% test prediction accuracy for all the genera were noted in our study*.*

**Fig. 6:**
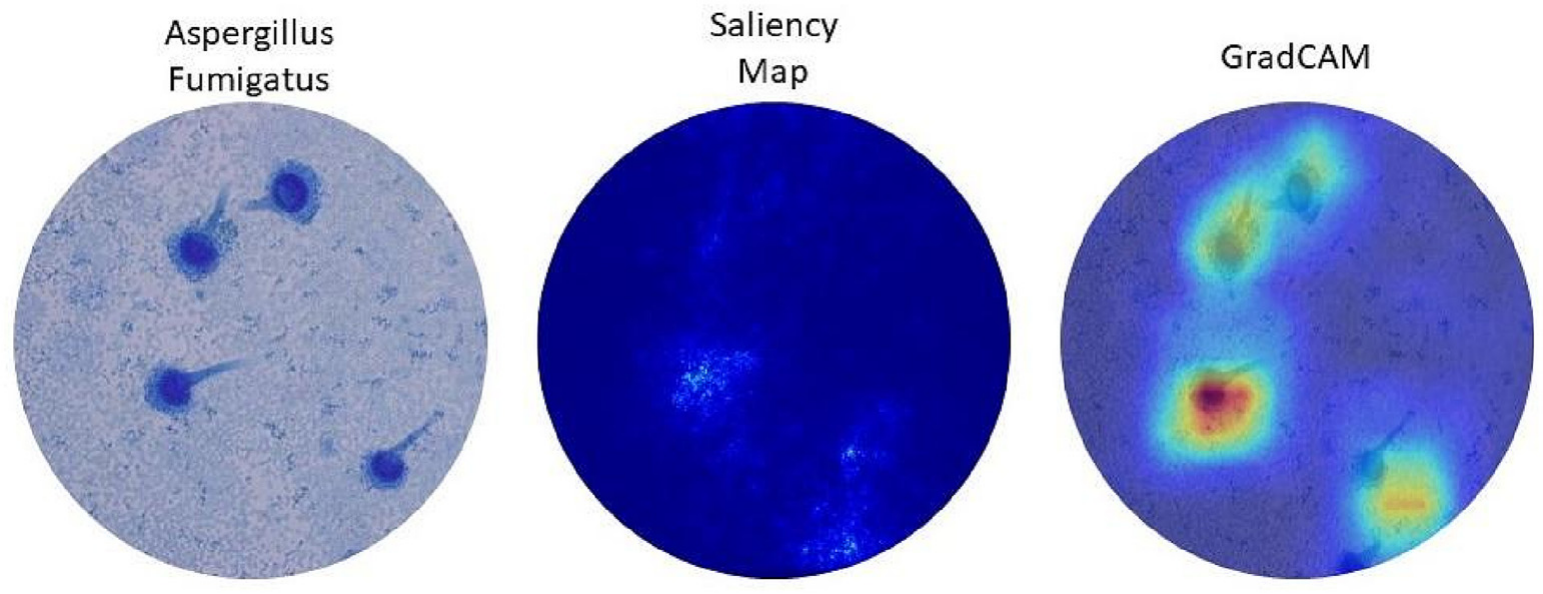
An example prediction result of our trained model (DenseNet) that shows image class, saliency map, and gradient class activation map (GradCAM).

In Table 2, a comparison of test prediction accuracy was shown between the augmented vs original data, ‘ImageNet’ pretrained vs Random weights initialization and Average vs Max pooling. It has been noted that the accuracy was almost 10%–15% higher for the augmented data. Also, it was significantly higher for the ImageNet pretrained models. Fig. 6 displays an example prediction result obtained with DenseNet model, backpropagated gradient saliency map,^29^, ^30^ and class activation heatmap.^31^, ^32^, ^36^ The saliency map and heatmap show areas in the image that made the most contribution to the output prediction.

**Table 2:** Test accuracy comparison between Augmented vs Original data, Random vs ImageNet pretrained weight initialization, and Average vs Max pooling.

Model	Training data		Weight initialization		Pooling	
	Original	Augmented	Random	ImageNet	Average	Max
DenseNet	45.70%	59.80%	50.50%	65.10%	59.80%	65.10%
Xception	43.70%	51.74%	48.60%	62.40%	51.74%	62.40%
Inception-ResNet	47.20%	58.70%	48.30%	59.30%	58.70%	59.30%
InceptionV3	50.10%	61.40%	37.80%	55.50%	61.40%	55.50%
ResNet50	53.20%	61.40%	39.50%	39.40%	61.40%	39.40%
VGG16	47.60%	49.61%	42.70%	55.60%	49.61%	55.60%
VGG19	41.70%	47.64%	29.40%	43.50%	41.70%	43.50%

Note: The accuracy results using Augmented vs Original data were statistically significant (*P* < .005). The accuracy results using Random vs ImageNet weights were statistically significant (*P* < .005). The accuracy results using Average vs Max pooling were not statistically significant (*P* > .005).

## Discussions

We presented predictions of filamentous fungi from culture using well-known deep learning architectures and identified the best model that shows promising results. As far as we know, this is the first study that has investigated a large number of pathogenic fungal genera (N=89) using deep learning. Prediction accuracy for the whole test dataset was low due to having a small number of sample images in some genera. There were 34 fungal genera that had less than 5 images. This results in a few samples in training and only 1 sample in validation and test set. Genera with larger numbers of images had better prediction accuracy. Almost all the prevalent or common genera in Fig 1 had at least 10 sample images in the training set. This might be the reason a better test accuracy was noted for those genera than the overall test samples. Apart from the common genera, we tested our model on the dataset provided by Zielinski^11^ and our model performed better than their model (average prediction accuracy of 96% vs 93.9%). In addition, our model did not require any extra pooling and classification methods compared to the model in Zielinski et al.^11^ as discussed in the introduction.

We noticed that the prediction accuracy increases by approximately 10% for most of the models when data augmentation was applied. This is a statistically significant increase in accuracy (*P* < .005). With augmentation, an increase of our training samples by a factor of 3 was achievable. As a result, each of our models were trained using more images and learned more features from those images. Hence, we accomplished better prediction accuracy on the test set. By data augmentation, we did not only improve our prediction accuracy but also made our model more robust for any unseen images. Apart from the ResNet50 model, there was no significant difference between Max pooling and Average pooling (*P* > .005). The training time was significantly higher for Average pooling compared to Max pooling. Our findings demonstrated that training our models with ImageNet pretrained network weights achieved better prediction results. The difference in prediction accuracy between ImageNet vs Random weights was statistically significant (*P* < .005). Also, the training time was shorter as the model started training from the initial weights closer to the optimal weights. Fine tuning was performed to see if prediction results could be improved, but was ultimately suboptimal compared to DenseNet without fine tuning. It seems that for these fungal images only the classification layer or last 2 layers were not sufficient enough to learn different features. Including more convolution and pooling layers at the top of the fine-tuning layers made the model perform with a greater degree of accuracy.

In our research, we noted promising results for some particular species but the overall performance across 89 species dropped due to the limited number of samples present in both training and test datasets for some genera, which is a limitation of this study. Increasing the number of sample images would improve the overall performance of our model. After implementation of improvements, this AI algorithm could potentially be used in clinical workflows to train inexperienced technologists and empower experienced ones with AI feedback in real time during microscopic examination.

## Conclusion

In summary, we present a deep learning approach model that shows promising results in prediction of filamentous fungi from culture, which could be used to increase diagnostic accuracy and decrease turnaround time. Inclusion of more sample images in the study set could improve prediction accuracy. The data and trained AI model used in this study could be shared with other researchers upon request.

## Declaration of interests

The authors declare that they have no known competing financial interests or personal relationships that could have appeared to influence the work reported in this paper.
